# Risk Related to Increasing Indications for Retrograde Intrarenal Surgery

**DOI:** 10.5152/tud.2024.23203

**Published:** 2024-01-01

**Authors:** Guglielmo Mantica, Francesca Ambrosini, Rafaela Malinaric, Alessandro Calarco, Carlo Terrone

**Affiliations:** 1 IRCCS Ospedale Policlinico San Martino, Genova, Italy; 2 Department of Surgical and Diagnostic Integrated Sciences (DISC), University of Genova, Genova, Italy; 3 Villa Pia Hospital, Rome, Italy

Dear Editor,

We read with interest the study of Tonyali et al,^1^ we commend the authors for the useful review, and we would like to provide our point of view regarding this topic, which is currently in the spotlight.

As many endourologists, we also believe that the technological advances, with new-generation high-power lasers and suctioning ureteral access sheath (UAS) already available in the market, might expand the indications for retrograde intrarenal surgery (RIRS) despite percutaneous nephrolithotomies (PCNL) and mini-PCNL. Clearly, RIRS represents the best minimally invasive treatment available, being able to be completely endoluminal, and for this reason, it would be the ideal treatment that we could offer to patients.^2^

However, although the advancement of RIRS over PCNL is desirable, there are some considerations that we believe must be taken into account.

The first is the simple fact that if RIRS will replace PCNL soon, it will probably be for medium size stones 15-30 mm, not for very complex staghorn stones. This fact could be highly detrimental in terms of training newbie endourologists. Training in endourology, like all surgery, works in steps: you move from seeing, to assisting, to performing simple cases, up to tackling very complex cases. It will be difficult for the new generation of endourologists to attempt percutaneous staghorn stones without taking advantage of numerous “simple” cases which will be carried out via RIRS. This can lead to a decrease in outcomes and an increase in complications, as well as a greater use of robotic surgery for very complex cases.^3^

The second reason is the possible increase in ureteral strictures associated with the use of the endoluminal route with UAS and high-power lasers.^4,5^ Ureteral access sheath placement carries an increased risk of ureteral wall ischemia and injury to the mucosal or muscular layers of the ureter, as well as a theoretical increased risk of ureteral strictures. Similarly, the use of high-power lasers for renal pelvic stones might increase the risk of ureteropelvic junction strictures.

In conclusion, RIRS represents the minimally invasive treatment par excellence in endourology, and its use and indications are likely to increase in the coming years. Nevertheless, we are probably not yet at the point of completely abandoning PCNL. It will be fundamental to invest in training and simulation to be able to carry out PCNL safely and with good outcomes for patients who cannot be managed with RIRS.
